# Beta diversity patterns of fish and conservation implications in the Luoxiao Mountains, China

**DOI:** 10.3897/zookeys.817.29337

**Published:** 2019-01-15

**Authors:** Jiajun Qin, Xiongjun Liu*, Yang Xu, Xaioping Wu, Shan Ouyang

**Affiliations:** 1 School of Life Sciences, Nanchang University, Nanchang 330031, China Nanchang University Nanchang China; 2 Key Laboratory of Poyang Lake Environment and Resource Utilization, Ministry of Education, School of Environmental and Chemical Engineering, Nanchang University, Nanchang 330031, China Nanchang University Nanchang China; 3 School of Resource, Environment and Chemical Engineering, Nanchang University, Nanchang 330031, China Nanchang University Nanchang China

**Keywords:** beta diversity, commercial fishes, Luoxiao Mountains, protected areas

## Abstract

The Luoxiao Mountains play an important role in maintaining and supplementing the fish diversity of the Yangtze River Basin, which is also a biodiversity hotspot in China. However, fish biodiversity has declined rapidly in this area as the result of human activities and the consequent environmental changes. Beta diversity was a key concept for understanding the ecosystem function and biodiversity conservation. Beta diversity patterns are evaluated and important information provided for protection and management of fish biodiversity in the Luoxiao Mountains. The results showed that the spatial turnover component was the main contributor to beta diversity of Hemiramphidae, Amblycipitidae, Catostomidae, Clariidae, Balitoridae and Percichthyidae in the Luoxiao Mountains, which indicated that a number of protected areas would be necessary to conserve fish biodiversity and that these families would need conservation measures. Most protected areas are currently limited to some regions; therefore, in order to protect fish diversity, conservation efforts must target an increase in the number of protected areas which should be spread across each of the regions.

## Introduction

Biodiversity patterns and their formation mechanisms have been one of the hot issues, and it is also an important foundation for conservation (Kennedy and Norman 2005; Sutherland et al. 2009). Biodiversity is important for the future sustainability of freshwater natural resources (Hiddink et al. 2008). While it is axiomatic that biodiversity is essential for sustainable productive fisheries there is surprisingly little supporting evidence (Dulvy et al. 2000; Hilborn et al. 2003). Freshwater fishes are among the most diverse assemblages on Earth, which provide important economic value (e.g., nutrition) and valuable ecosystem services (e.g., natural water filtration; Naylor et al. 2000; Cressey 2009; De Silva 2012). However, due to dam construction, overfishing (commercial fish fishing), pollution, deforestation, and other human activities, fish numbers have declined rapidly in global terms (Fu et al. 2003; Arthington et al. 2016; Liu et al. 2017) and they are thus one of the most threatened assemblages.

Beta diversity is an important tool for conservation planning (Anderson et al. 2006); knowledge on beta diversity patterns can aid the decision on the number of protected areas needed and their sizes (Margules and Pressey 2000; Wiersma and Urban 2005). Beta diversity can be decomposed into species turnover (species replacement) and nestedness (richness difference; Baselga 2010; Carvalho et al. 2012). The species turnover component (species replacement) is the replacement of some species by others leading to a low number of shared species among two communities where turnover is high (Baselga 2010). In addition, the nestedness component (richness difference components) represents the differences between two communities only in terms of species richness, with the poorer community as a subset of the richer one (Baselga 2010). According to the percentage of spatial turnover and nestedness components in total beta diversity, different conservation strategies can be selected. If species turnover is the main component of beta diversity, a larger number of protected areas would be necessary to conserve regional biodiversity (Baselga 2010; Carvalho et al. 2012). If the nestedness is the main component of beta diversity, one large protected area comprising a high species richness could be sufficient (Baselga 2010; Carvalho et al. 2012).

The Luoxiao Mountains range is located in the southeast of China’s mainland and has a long history and complex environmental factors (Liao et al. 2014; Wei et al. 2015). The northern part of the mountains is connected with the Yangtze River, and the southern part is connected with the Nanling Mountain (Gong et al. 2016). It is the most important ecotone and fragile zone in the third step of eastern China, and is an important channel for the migration and diffusion of terrestrial organisms in the Northern Hemisphere (Liao et al. 2014; Gong et al. 2016). In addition, the Luoxiao Mountains is also a biodiversity hotspot in China (Liao et al. 2014; Gong et al. 2016). At the same time, as being the watershed of the Poyang Lake Basin and the Dongting Lake Basin in the middle reaches of the Yangtze River, the Luoxiao Mountains are a refuge to many endemic and endangered fishes (Liao et al. 2014; Gong et al. 2016). Therefore, fish resources of the Luoxiao Mountains play an important role in maintaining and supplementing the aquatic biodiversity of the Yangtze River Basin. However, due to dam construction, overfishing, pollution, deforestation, and other human activities, fish diversity declined rapidly in this region. Here, we aim to evaluate beta diversity patterns and to provide useful information for the protection and management of fish biodiversity in the Luoxiao Mountains.

## Material and methods

### Study area

The Luoxiao Mountains (25°32'–29°28'N, 113°09'–114°26'E) are a large system of mountain ranges, located in the southeast of China’s mainland with an overall north-south trend, stretching across Hubei, Hunan, and Jiangxi provinces. It consists of Mufu Mountain, Jiuling Mountain, Wugong Mountain, Zhuguang Mountain, and others. The total length of the Luoxiao Mountains is 400 km and altitude ranges are 82–2120 m. Lingfeng Peak (2122 m) is one of the highest mountains in the southeastern Eurasia. Its average precipitation range is 1341–1943 mm and forest coverage in the watershed reaches 90% (Table 1). The tributaries of the Ganjiang River from the eastern stream of the Luoxiao Mountains flow into Poyang Lake. The tributaries of Xiangjiang River from the western stream of it flow into the Dongting Lake. The Fushui River alone flows into the Yangtze River.

**Table 1.** Hydrology and environmental characteristics of the streams of Luoxiao Mountains. JJ: Jinjiang River; YS: Yuanshui River; HS: Heshui River; SS: Shushui River; SC: Suichuan River; SY: Shangyou River; MS: Mishui River; ML: Miluo River; FS: Fushui River; XH: Xiuhe River; LY: Liuyang River.

**Stream**	**Latitude**	**Longitude**	**Length (km)**	**Area (km^2^)**	**Average gradient (%)**	**Average precipitation (mm)**	**Average temperature (°C)**	**Annual average runoff (×10^8^ m^3^)**	**Average Altitude (m)**
LY	28°24'–28°46'	112°99'–114°04'	222	4665	0.57	1598	17.3	39.41	252
MS	27°16'–26°25'	112°88'–113°99'	296	10305	1.01	1483	18.1	76.03	352
ML	28°86'–29°02'	112°93'–114°05'	253	5543	0.46	1400	17.6	43.04	250
FS	29°49'–29°86'	114°40'–115°45'	196	5250	0.79	1275	16.6	43.5	613
XH	28°31'–29°12'	114°14'–116°01'	419	14700	0.48	1663	16.7	135.1	676
SY	25°37’-25°49’	113°43'–114°49'	204	4647	0.70	1570	18.8	33	615
SC	26°11'–26°30'	113°56'–114°44'	176	2882	2.36	1640	16.9	27.1	971
SS	126°29'–26°47'	14°04'–114°50'	152	1301	2.14	1630	16.7	11.3	1610
HS	27°04'–27°24'	114°01'–114°59'	256	9103	0.59	1580	17.8	27.4	747
YS	27°27'–28°04'	114°10'–115°29'	279	6262	0.34	1678	17.2	29.6	391
JJ	27°57'–28°25'	114°01'–115°49'	307	7886	0.26	1679	17.6	70	391

### Sampling methods

**Figure 1.**
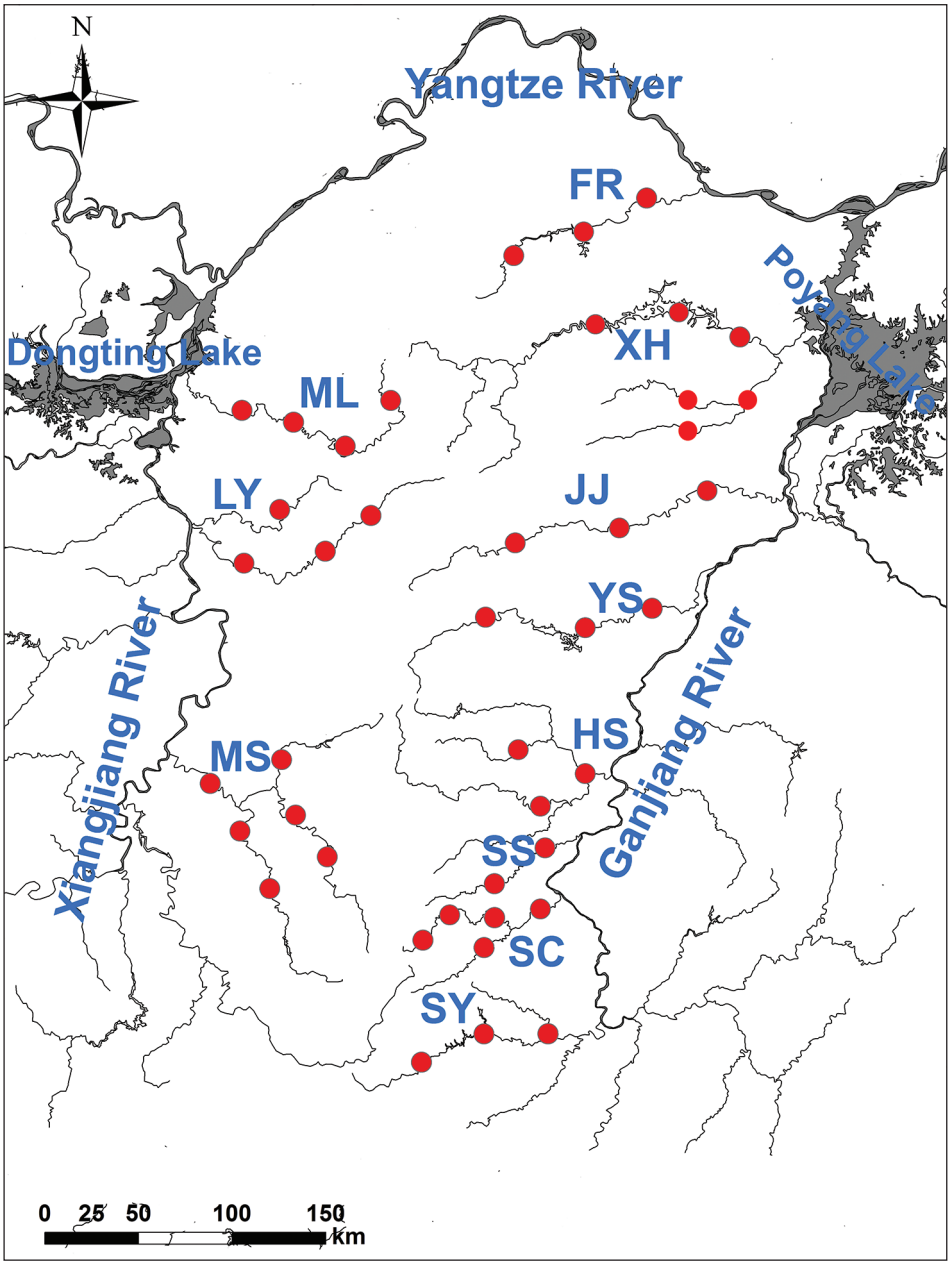
Map showing the sampling location of the streams of the Luoxiao Mountains.

Sampling sites were selected by considering habitats, variations, and anthropogenic activities in the Luoxiao Mountains. Fish samples were collected from April 2014 to 2017 in eleven streams of the Luoxiao Mountains. We selected eleven streams (42 sampling sites) (Figure 1), including the (1) Fu River (sampling code is FR; three sampling sites), Xiuhe River (sampling code is XH; six sampling sites), Jinjiang River (sampling code is JJ; three sampling sites), Yuanshui River (sampling code is YS; three sampling sites), Heshui River (sampling code is HS; three sampling sites), Shushui River (sampling code is SS; two sampling sites), Suichuan River (sampling code is SC; five sampling sites), Shangyou River (sampling code is SY; three sampling sites), Miluo River (sampling code is ML; four sampling sites), Liuyang River (sampling code is LY; four sampling sites), Mishui River (sampling code is MS; six sampling sites). We collected the fish catch from professional fishermen who captured fish using fully standardized five gillnet clusters, each consisting of six gillnets of 50–80 m in length 4–10 m in height (mesh size = 1.0–10.0 cm) in the Luoxiao Mountains rivers. In addition, we assumed similar capture efficiencies from gillnet samples at each site. At the same time, we surveyed and collected fish in the township markets along the river which enhanced the species checklists at each section. All fish specimens were identified according to Chen (1998), Chu et al. (1999), and Yue (2000), and the scientific name was corrected according to Fishbase (http://www.fishbase.org/search.php). The division of endangered categories of fish was decided according to Jiang et al. (2016) and IUCN (2017).

### Data analysis

Beta diversity is represented by the difference in species composition between different communities, which was determined by species turnover (species replacement) and nestedness (richness difference; Baselga 2010; Carvalho et al. 2012). In order to quantify the effects of two processes, Baselga (2010) systematically proposed the beta diversity decomposition method (BAS frameworks) based on the Sørensen index (β_sor_), which was decomposed into species spatial turnover components (β_sim_) and nestedness components (β_sne_). Podani and Schmera (2011) and Carvalho et al. (2012) proposed the beta diversity decomposition method (POD frameworks) based on the Jaccard index (β_jac_), which was decomposed into species replacement components (β_-3_) and richness difference components (β_rich_). Here, we analyzed the fish biodiversity based on both the BAS and POD frameworks.

BAS frameworks (Sørensen index):


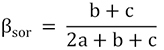



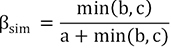



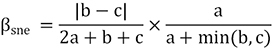


POD frameworks (Jaccard index):


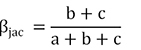



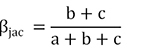



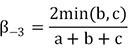


## Results

### Fish species composition

**Figure 2.**
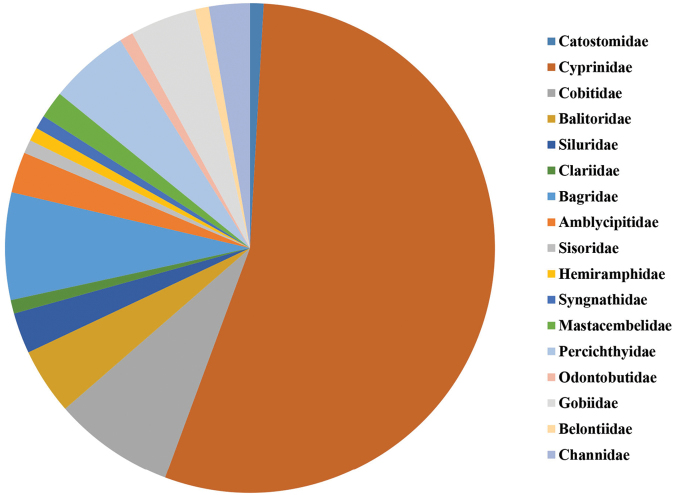
Fish composition from the streams of the Luoxiao Mountains.

The fish specimens sampled and identified in the Luoxiao Mountains were categorized into 113 species and 17 families (Figure 2; Appendix 2). The number of Cypriniformes was the greatest, accounting for 68.1% of the total number of fish species, followed by Siluriformes and Perciformes, accounting for 14.2% each, and Beloniformes, accounting for 0.1% (Figure 2). In addition, according to the endangered categories of the Jiang et al. (2016), Least Concern fish species were the greatest, accounting for 77.9% of the catch (Appendix 3). Critically Endangered, Vulnerable, and Near Threatened fish species accounted for 7.1% (Appendix 3).

### Beta diversity patterns

The fish composition similarity in the Luoxiao Mountains had a mean value of 0.50 and 0.67, based on BAS and POD frameworks respectively (SD ± 0.06 and SD ± 0.05, respectively; Table 2). The spatial turnover and replacement components (β_sim_ and β_-3_, 0.36 ± 0.08, and 0.39 ± 0.13) were greater than its nestedness and richness difference components (β_sne_ and β_Rich_, 0.14 ± 0.09 and 0.28 ± 0.16). FS and SC had a high β_sor_ and β_jac_ (0.55 ± 0.07 and 0.54 ± 0.11; 0.71 ± 0.05 and 0.69 ± 0.09), a high spatial turnover and replacement components (0.39 ± 0.07 and 0.45 ± 0.12) in LY and nestedness and richness difference components (0.22 ± 0.10 and 0.41 ± 0.17) in SC (Table 2).

**Table 2.** Fish compositional similarity by BAS and POD frameworks in the streams of Luoxiao Mountains. JJ: Jinjiang River; YS: Yuanshui River; HS: Heshui River; SS: Shushui River; SC: Suichuan River; SY: Shangyou River; MS: Mishui River; ML: Miluo River; FS: Fushui River; XH: Xiuhe River; LY: Liuyang River; ES: Eastern stream of Luoxiao Mountain; WS: Western stream of Luoxiao Mountains.

**Stream**	**β**					
	**BAS**			**POD**		
	**β_sor_**	**β_sim_**	**β_sne_**	**β_jac_**	**β_-3_**	**β_rich_**
JJ	0.52±0.08	0.32±0.11	0.19±0.13	0.68±0.07	0.33±0.16	0.35±0.19
YS	0.48±0.08	0.27±0.06	0.21±0.11	0.65±0.07	0.27±0.11	0.37±0.17
HS	0.52±0.08	0.39±0.09	0.13±0.09	0.68±0.07	0.42±0.14	0.26±0.16
SS	0.49±0.07	0.38±0.07	0.11±0.07	0.66±0.06	0.42±0.12	0.24±0.14
SC	0.54±0.11	0.31±0.09	0.22±0.10	0.69±0.09	0.28±0.11	0.41±0.17
SY	0.48±0.03	0.37±0.10	0.11±0.09	0.65±0.03	0.42±0.15	0.22±0.14
MS	0.47±0.05	0.36±0.06	0.11±0.08	0.64±0.05	0.41±0.12	0.22±0.14
ML	0.50±0.04	0.38±0.10	0.12±0.09	0.66±0.04	0.42±0.14	0.24±0.15
FS	0.55±0.07	0.39±0.06	0.16±0.10	0.71±0.05	0.39±0.15	0.32±0.19
XH	0.48±0.04	0.36±0.08	0.11±0.08	0.65±0.03	0.42±0.14	0.23±0.15
LY	0.49±0.03	0.39±0.07	0.10±0.07	0.66±0.03	0.45±0.12	0.21±0.12
ES	0.50±0.07	0.34±0.09	0.16±0.11	0.66±0.06	0.36±0.14	0.30±0.17
WS	0.50±0.06	0.38±0.07	0.12±0.09	0.67±0.05	0.42±0.13	0.25±0.15
Total	0.50±0.06	0.36±0.08	0.14±0.09	0.67±0.05	0.39±0.13	0.28±0.16

At the same time, fish composition similarity (β_sør_ and β_jac_) for the entire fish fauna had a mean value of 0.66 and 0.76 (SD ± 0.24 and 0.21, Table 3). The spatial turnover and replacement components (β_sim_ and β_-3_, 0.41 ± 0.03 and 0.32&nbsp;± 0.03) were higher than the nestedness and richness difference components (β_sne_ and β_rich_, 0.25 ± 0.02 and 0.44 ± 0.02). The greatest β_sor_ and β_jac_ (0.93±0.16 and 0.96±0.13) and spatial turnover and replacement components (0.80 ± 0.04 and 0.49 ± 0.03) was in Hemiramphidae, followed by Amblycipitidae, and the lowest was in Syngnathidae. The greatest nestedness and richness difference components (0.53&nbsp;±&nbsp;0.25 and 0.65 ± 0.26) was in Syngnathidae, followed by Siluridae, and the lowest was in Hemiramphidae (Table 3).

**Table 3.** BAS and POD frameworks based on all species and 17 families in the streams of Luoxiao Mountain. Values are mean ± standard deviation.

**Family**	**BAS**			**POD**		
	**β_sor_**	**β_sim_**	**β_sne_**	**β_jac_**	**β_-3_**	**β_rich_**
Catostomidae	0.70±0.32	0.39±0.05	0.31±0.03	0.77±0.30	0.25±0.03	0.51±0.03
Cyprinidae	0.65±0.24	0.41±0.03	0.25±0.02	0.76±0.20	0.32±0.02	0.43±0.02
Cobitidae	0.66±0.26	0.37±0.04	0.28±0.03	0.75±0.23	0.28±0.03	0.47±0.03
Balitoridae	0.69±0.25	0.49±0.04	0.20±0.02	0.78±0.21	0.39±0.03	0.39±0.02
Siluridae	0.64±0.30	0.26±0.03	0.38±0.03	0.73±0.29	0.17±0.02	0.56±0.03
Clariidae	0.70±0.32	0.39±0.05	0.31±0.03	0.77±0.30	0.25±0.03	0.51±0.03
Bagridae	0.67±0.24	0.41±0.03	0.27±0.02	0.77±0.21	0.32±0.03	0.45±0.03
Amblycipitidae	0.78±0.23	0.58±0.04	0.21±0.03	0.85±0.19	0.40±0.03	0.45±0.03
Sisoridae	0.56±0.22	0.34±0.03	0.22±0.02	0.69±0.18	0.31±0.03	0.38±0.02
Hemiramphidae	0.93±0.16	0.80±0.04	0.14±0.03	0.96±0.13	0.49±0.03	0.47±0.03
Syngnathidae	0.53±0.25	0	0.53±0.25	0.65±0.26	0	0.65±0.26
Mastacembelidae	0.64±0.22	0.43±0.03	0.21±0.02	0.75±0.18	0.38±0.03	0.37±0.02
Percichthyidae	0.69±0.24	0.49±0.04	0.20±0.02	0.78±0.20	0.40±0.03	0.39±0.02
Odontobutidae	0.65±0.25	0.49±0.04	0.16±0.02	0.76±0.20	0.33±0.16	0.35±0.19
Gobiidae	0.66±0.25	0.42±0.04	0.24±0.02	0.76±0.22	0.32±0.03	0.44±0.02
Belontiidae	0.53±0.23	0.31±0.03	0.23±0.02	0.67±0.20	0.28±0.03	0.39±0.02
Channidae	0.58±0.22	0.35±0.03	0.23±0.02	0.71±0.19	0.30±0.02	0.41±0.02
All species	0.66±0.24	0.41±0.03	0.25±0.02	0.76±0.21	0.32±0.03	0.44±0.02

**Figure 3.**
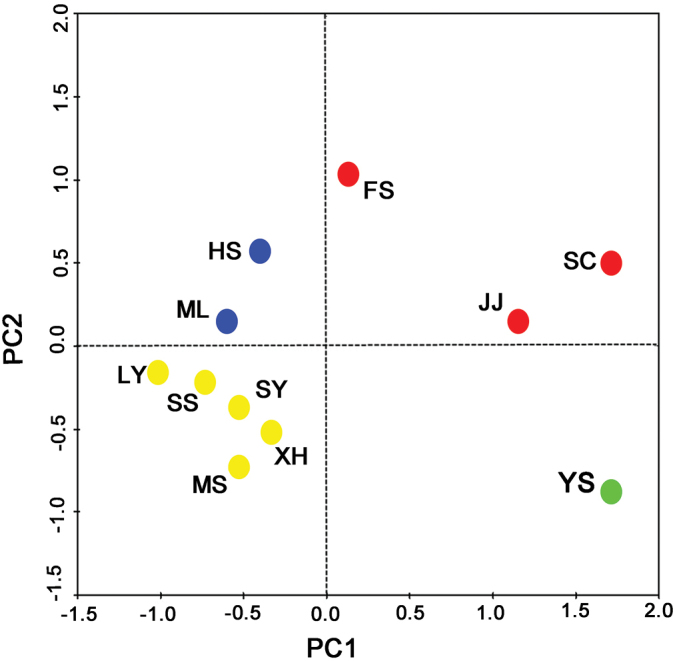
Results of the principal component analysis (PCA) on the compositional similarity of fish species in the streams of the Luoxiao Mountains. JJ: Jinjiang River; YS: Yuanshui River; HS: Heshui River; SS: Shushui River; SC: Suichuan River; SY: Shangyou River; MS: Mishui River; ML: Miluo River; FS: Fushui River; XH: Xiuhe River; LY: Liuyang River.

The PCA showed that fish composition similarity of LY, SS, SY, XH, and MS were similar based on BAS and POD frameworks; FS, JJ and SC were similar; HS and ML were similar; and YS was uniquely divided into other areas, respectively (Figure 3).

We found almost no significant effects of geographical drivers on overall beta diversity for the Luoxiao Mountains (Table 4). The correlation between BAS and POD frameworks and difference in length, average precipitation, mean temperature, and average gradient were not significant in the Luoxiao Mountains. The correlation between β_sne_ (β_rich_) and differences in area and annual average runoff was significant. The correlation between β_sim_ (β_-3_) and difference in average altitude was also significant (Table 4).

**Table 4.** Effects of geographical drivers on pairwise compositional similarity and its partitioned components obtained from BAS and POD frameworks in the streams of Luoxiao Mountain, Jiangxi Province. Significant results (P < 0.05) are in bold.

			**Area**	**Length**	**Average precipitation**	**Annual average runoff**	**Average altitude**	**Average gradient**	**Average temperature**
BAS	β_sor_	*r*	0.162	0.134	-0.008	0.273	0.055	-0.162	-0.070
		*P*	0.190	0.203	0.454	0.069	0.333	0.850	0.647
	β_sim_	*r*	-0.318	-0.245	0.187	-0.289	0.248	0.128	0.058
		*P*	0.931	0.871	0.127	0.888	**0.032**	0.272	0.415
	β_sne_	*r*	0.386	0.304	-0.168	0.436	-0.179	-0.221	-0.098
		*P*	**0.022**	0.062	0.805	**0.011**	0.861	0.943	0.658
POD	β_jac_	*r*	0.155	0.122	-0.017	0.254	0.067	-0.147	-0.076
		*P*	0.150	0.244	0.521	0.072	0.328	0.808	0.680
	β_-3_	*r*	-0.366	-0.285	0.164	-0.401	0.236	0.219	0.081
		*P*	0.961	0.913	0.160	0.970	**0.028**	0.051	0.370
	β_rich_	*r*	0.365	0.284	-0.145	0.430	-0.176	-0.237	-0.096
		*P*	**0.019**	0.076	0.758	**0.015**	0.851	0.981	0.630

## Discussion

### Fish species composition

Studies on fish composition and diversity in streams is the basis for the conservation and management of stream fishes (Liu et al. 2017; Zhang et al. 2018). In this study, the fish specimens sampled and classified in the stream of the Luoxiao Mountains were categorized into 113 species. Compared with species numbers of the Shiwanda Mountains (102 species; Zhao and Zhang 2001), Wuyi Mountains (117 species; Song et al. 2017), and the Tibetan Plateau (114 species; Wu and Tan 1991), the fish abundance in the Luoxiao Mountains was also higher.

### Beta diversity patterns

Abiotic and biotic factors and their ecological processes in different stream sizes varies substantially (Zhang et al. 2018). At least in streams, local species richness of fishes, habitat diversity and complexity often increase in large streams (Roberts and Hitt 2010; Zhang et al. 2018). Comparing alpha diversity and beta diversity at local and landscape scales is an important, yet little-understood, area of basic and applied ecological research (Kessler et al. 2009). However, most studies on fish diversity of streams have focused on alpha diversity, whereas fewer studies have investigated beta diversity (Tisseuil et al. 2013; Johnson and Angeler 2014). Knowledge of beta diversity patterns can go beyond the systematic conservation planning method that only considers the location of protected area in relation to natural physical and biological patterns (Margules and Pressey 2000; Wiersma and Urban 2005). The efficiency of protected areas not only relies on species richness, but also on how well the complementarity among sites increases biodiversity conservation (Howard et al. 1998; Bush et al. 2016; Socolar et al. 2016). In this study, as turnover brought the larger contribution to beta diversity, additional conservation efforts must target an increase in the number of protected areas, which should be spread across each one of the regions, to maximize the protection of species diversity.

### Biogeographical processes

The modern freshwater fish fauna of Eurasia originated in the early Tertiary (Chen et al. 1986; Liu and Quan 1996; Zhang 2012). At the same time, the primitive species of the Danioninae and Barbinae became the main component of the fish fauna with the flattened land and the warming climate (Chen et al. 1986; Tang et al. 2001). During the dramatic changes of landscape and climate of the Eurasian continent in the late Oligocene and the end of the Pliocene, the primitive species component had been reduced rapidly (Chen et al. 1986; Tang et al. 2001). After the Quaternary ice age, only some offspring fishes of the old Tertiary Period remained (Chen et al. 1986; Tang et al. 2001). Moreover, Labeoninae, Gastromyzontidae, Balitoridae and Sisoridae were dominant during the uplift of the Tibetan Plateau (Chen et al. 1986; Yang et al. 1982; Tang et al. 2001). At the same time, a large area of alluvial plains appeared in eastern China, and special habitats were created under the influence of the East Asian monsoon (Chen et al. 1986; Zhang and Chen 1997). The cold-water fishes, such as Leuciscinae and Gobioninae became the endemic fishes of the river plain in East Asia (Hypophthalmichthyinae, Culterinae, Xenocyprininae, Acheilognathinae, Gobiobotinae) and the warm-water fishes the endemic fishes of Southeast Asia (Botiinae, Clariidae, Amblycipitidae, Belontiidae, Channidae, Mastacembelidae). Since then, these taxa have become the major faunal component in southern China (Chen et al. 1986). In this study, Culterinae, Gobioninae, and Acheilognathinae had a high species composition (Appendix 2). At the same time, the spatial turnover component is the main contributor of beta diversity in Hemiramphidae, Amblycipitidae, Catostomidae, Clariidae, Balitoridae, and Percichthyidae, indicating that it would be necessary to conserve habitats in the Luoxiao Mountains.

### Threats to fish diversity

The headwater stream is a tributary of a larger river, which is often located in a mountainous area with high altitude. Compared with large rivers, it had relatively simple habitat structure, poor nutrition, obvious hydrological change, and low species diversity (Vannote et al. 1980; Grossman et al. 1990; Zhang et al. 2018). Therefore, the ecosystem of the stream is more fragile, its resistance to external disturbance and resilience is lower, and it would be more difficult to recover once it is damaged by humans. Fish, as the apex consumers of the stream, are very important to the stability and functioning of the stream ecosystems (Nogueira et al. 2010; Yan et al., 2011; Arthington et al. 2016; Liu et al. 2017). During the long evolution process, fishes have gradually adjusted their corresponding morphological characteristics, phenological rhythms, and life history countermeasures so that they could adapt to the unique natural environment of the stream (Lytle and Poff 2004; Osorio et al. 2011; Ren et al. 2016). However, due to habitat loss, water pollution, alien-species invasions, forest overcutting, climate change, overfishing etc., the fish biodiversity of most streams in China have been seriously threatened (Dudgeon et al. 2006; Allan and Castillo 2007). For example, numerous small dams in mountain streams were established (Huang et al. 2008; Hu et al. 2009). Dam constructions modified these small fast-flowing streams, which led to the decline of fish species adapted to rapid streams (Hu et al. 2009). In addition, a large number of fishing methods such as traps, gill nets, and electro-fishing has led to overfishing which has also caused a dramatic decline in fish biodiversity (Huang and Gong 2007; Zhang et al. 2010). Heavy metal pollution has affected the aquatic ecosystem in the Luoxiao Mountains (He et al. 1998). The contents of heavy metals have greatly exceeded the recommended standards (Xu et al. 2016). In this study, critically endangered (*Myxocyprinusasiaticus*), vulnerable (*Leptobotiaelongata*, *Pseudobagruspratti*, *Liobagrusmarginatus*, *Sinipercaroulei*), and near threatened (*Onychostomabarbatulum*, *Sinipercaobscura*, *Sinipercaundulata*) fish species accounted for 7.1% of the species recovered. At the same time, the PCA results showed that the fish composition among the streams sampled in the Luoxiao Mountains were similar. As turnover brought the larger contribution to beta diversity, additional conservation efforts must target an increase in the number of protected areas, which should be spread across each of the regions, to maximize the protection of species diversity.

### Conservation implications

Freshwater fishes were thought to be the world’s most threatened group of vertebrates after amphibians (Bruton 1995; Hiddink et al. 2008; Liu et al. 2017) and, without protection, 20% of the world’s freshwater fishes may become extinct in the next 50 years (Moyle and Leidy 1992; Fu et al. 2003). Although endangered fish have raised public awareness, conservation strategies of fish biodiversity in China are concentrated on endangered species and economic fish (Fu et al. 2003; Liu et al. 2017). In addition, protected areas mainly occur in terrestrial conservation strategies, but freshwater habitats are commonly protected only incidentally as part of their inclusion within terrestrial reserves (Huang et al. 2013). For example, conservation areas of plants, animals, and wetlands in Jiangxi Province have been established, but there are very few freshwater protected areas nor are there any fish passage facilities in the rivers (Huang et al. 2013). In this study, species turnover component is the main pattern of beta diversity, implying that a larger number of protected areas would be necessary to conserve the regional biodiversity in the Luoxiao Mountains. Therefore, in order to protect fish biodiversity, the establishment of freshwater protected areas in the streams of the Luoxiao Mountains should be considered.

## Appendix 1

**Table:** Species occurrence in the streams of Luoxiao Mountains. 1 = presence of the species as native in the stream, 0 = the species is absent from the stream, 2 = the species is present in the stream, but non-native from this stream. JJ: Jinjiang River; YS: Yuanshui River; HS: Heshui River; SS: Shushui River; SC: Suichuan River; SY: Shangyou River; MS: Mishui River; ML: Miluo River; FS: Fushui River; XH: Xiuhe River; LY: Liuyang River.

*Species*	JJ	YS	HS	SS	SC	SY	MS	ML	FS	XH	LY
* Myxocyprinus asiaticus *	0	0	0	0	1	0	0	0	0	0	0
* Zacco platypus *	1	1	1	1	1	1	1	1	1	1	1
* Opsariichthys bidens *	1	1	1	1	1	1	1	1	1	1	1
* Mylopharyngododon piceus *	0	1	0	0	0	1	1	0	0	0	1
* Ctenopharyngodon idella *	1	1	0	0	1	1	1	0	1	1	0
* Elopichthys bambusa *	0	0	0	0	0	0	0	0	0	0	1
* Squaliobarbus curriculus *	0	1	0	0	1	0	0	0	0	0	1
* Hemiculter leucisculus *	1	1	1	1	1	1	0	0	0	0	1
* Hemiculter bleekeri *	0	1	0	1	1	1	0	0	0	1	0
* Hemiculterella sauvagei *	1	1	1	0	0	0	0	0	0	0	0
* Hemiculterella wui *	0	0	0	0	1	1	0	1	0	1	0
* Pseudohemiculter dispar *	1	1	1	1	1	1	1	1	1	1	1
* Pseudolaubuca sinensis *	0	0	0	0	1	0	0	0	0	0	0
* Sinibrama macrops *	1	1	0	0	1	1	1	1	0	1	0
* Chanodichthys erythropterus *	0	0	1	0	0	0	0	0	1	1	1
* Culter alburnus *	0	1	0	1	0	1	1	0	1	0	0
* Chanodichthys mongolicus *	0	0	1	0	0	0	0	0	0	0	1
* Chanodichthys dabryi *	0	1	0	1	0	0	0	0	1	0	1
* Culter oxycephaloides *	0	0	0	0	0	0	0	0	0	1	1
* Parabramis pekinensis *	0	0	0	0	1	1	1	1	0	0	0
* Megalobrama terminalis *	0	0	0	0	0	0	0	0	0	1	1
* Megalobrama amblycephala *	0	0	0	1	1	0	0	0	1	0	0
* Xenocypris macrolepis *	0	1	0	1	1	1	0	0	0	1	0
* Xenocypris davidi *	1	1	1	1	0	0	0	0	0	1	1
* Plagiognathops microlepis *	0	1	0	0	0	0	0	0	0	0	1
* Distoechodon tumirostris *	1	0	0	0	0	0	0	0	1	1	0
* Hypophthalmichthys molitrix *	0	1	0	1	1	1	0	0	0	0	1
* Hypophthalmichthys nobilis *	0	1	0	0	1	1	0	0	0	0	1
* Abbottina rivularis *	0	1	1	1	1	0	1	1	0	1	1
* Pseudorasbora parva *	1	1	0	1	0	0	1	0	1	0	1
* Pseudogobio vaillanti *	0	1	0	0	0	0	0	0	0	0	0
* Pseudogobio guilinensis *	0	0	0	0	1	0	0	0	0	0	0
* Hemibarbus labeo *	0	1	0	0	1	0	0	0	0	0	0
* Hemibarbus maculatus *	0	1	0	0	1	1	0	1	0	0	1
* Huigobio chenhsienensis *	0	1	0	0	1	0	0	0	0	0	0
* Sarcocheilichthys sinensis *	1	1	0	0	1	0	0	0	0	1	1
* Sarcocheilichthys kiangsiensis *	0	1	0	0	0	0	0	0	0	1	0
* Sarcocheilichthys nigripinnis *	0	0	0	0	0	0	1	0	0	1	1
* Squalidus argentatus *	0	1	1	1	1	1	1	0	1	0	1
* Rhinogobio typus *	1	0	1	0	0	0	1	0	0	1	1
* Platysmacheilus exiguus *	0	0	0	1	1	0	0	0	0	0	0
* Saurogobio dabryi *	1	1	1	0	1	1	1	1	0	1	1
* Saurogobio xiangjiangensis *	0	0	0	0	1	0	0	0	0	0	1
* Microphysogobio kiatingensis *	0	0	0	1	1	0	0	0	0	0	0
* Microphysogobio fukiensis *	0	0	0	1	0	0	1	0	0	1	0
* Gobiobotia filifer *	0	0	0	0	1	1	0	0	0	0	0
* Gobiobotia longibarba *	0	0	0	0	0	0	0	1	0	0	0
* Acheilognathus macropterus *	0	0	0	1	1	0	0	0	0	0	1
* Acheilognathus gracilis *	0	1	0	1	1	0	0	0	0	1	1
* Acheilognathus chankaensis *	0	0	0	1	0	1	0	1	0	0	0
* Acheilognathus tonkinensis *	0	0	0	0	0	0	1	1	1	0	1
* Acheilognathus barbatulus *	0	1	0	0	0	0	0	0	0	0	0
* Rhodeus ocellatus *	0	1	0	1	1	1	1	1	1	1	1
* Rhodeus lighti *	0	0	0	0	0	0	0	1	1	0	0
* Acrossocheilus fasciatus *	0	1	1	1	0	0	1	0	0	1	0
* Acrossocheilus paradoxus *	0	0	0	1	1	0	1	0	0	0	0
* Acrossocheilus hemispinus *	0	0	1	0	0	0	1	1	0	0	0
* Acrossocheilus parallens *	0	1	1	1	1	1	1	1	0	1	1
* Spinibarbus hollandi *	0	1	1	0	1	1	0	0	0	0	0
* Onychostoma barbatulum *	0	1	0	0	0	0	0	0	0	0	0
* Carassius auratus *	1	1	1	1	1	1	1	1	1	1	1
* Cyprinus carpio *	1	1	1	1	1	1	1	1	1	1	1
* Garra orientalis *	0	0	0	0	1	0	0	0	0	1	0
* Cobitis sinensis *	0	1	0	0	1	0	0	1	1	1	0
* Misgurnus anguillicaudatus *	1	1	1	1	1	1	1	1	1	1	1
* Paramisgurnus dabryanus *	0	1	0	1	1	0	0	0	0	0	0
* Schistura fasciolata *	0	1	0	0	0	0	0	0	0	0	0
* Schistura incerta *	0	0	1	0	0	0	0	0	0	0	0
* Leptobotia elongata *	0	0	0	0	1	0	0	0	0	0	0
* Parabotia banarescui *	0	0	0	0	1	0	0	0	0	0	0
* Parabotia fasciata *	0	1	0	0	1	0	0	0	0	0	0
* Parabotia maculosa *	0	1	0	0	1	0	0	0	0	0	0
* Erromyzon sinensis *	0	0	0	0	1	0	0	0	0	0	0
* Lepturichthys fimbriata *	0	0	0	1	1	0	1	0	0	0	0
* Vanmanenia stenosoma *	0	0	0	1	1	0	0	0	0	1	0
* Vanmanenia pingchowensis *	0	1	0	1	0	0	0	0	0	0	0
* Pseudogastromyzon changtingensis *	0	0	1	1	0	0	0	0	0	0	0
* Silurus asotus *	1	1	1	1	1	1	1	1	1	1	1
* Silurus meridionalis *	0	0	0	0	1	0	0	0	0	0	0
* Pterocryptis cochinchinensis *	0	0	0	0	1	0	0	0	0	0	0
* Clarias fuscus *	0	0	0	0	1	0	0	0	0	0	0
* Hemibagrus macropterus *	0	0	0	0	1	0	1	1	0	0	1
* Pseudobagrus crassilabris *	0	0	0	0	1	0	1	0	0	0	0
* Pseudobagrus tenuis *	0	1	0	1	1	0	1	1	0	1	0
* Pseudobagrus ondon *	0	1	0	0	0	0	1	0	0	0	0
* Pseudobagrus pratti *	0	0	0	0	1	0	0	0	0	0	0
* Pseudobagrus albomarginatus *	0	0	0	0	0	0	0	0	0	1	0
* Tachysurus fulvidraco *	1	1	1	1	1	1	1	1	1	1	1
* Tachysurus nitidus *	0	0	0	0	0	0	1	0	0	1	0
* Liobagrus anguillicauda *	0	0	1	0	0	0	0	0	0	0	0
* Liobagrus marginatus *	0	1	0	0	0	0	0	0	0	0	0
* Liobagrus nigricauda *	0	1	1	1	0	0	0	1	0	0	0
* Glyptothorax sinense *	0	1	0	1	1	0	0	1	0	0	0
* Hyporhamphus intermedius *	0	0	0	0	0	0	0	0	1	0	0
* Monopterus albus *	1	1	1	1	1	1	1	1	1	1	1
* Macrognathus aculeatus *	0	1	0	0	0	0	1	0	0	0	0
* Sinobdella sinensis *	0	1	0	0	1	0	0	1	0	1	0
* Siniperca chuatsi *	0	1	0	0	1	0	1	0	0	1	0
* Siniperca knerii *	0	1	0	0	1	0	0	0	0	0	0
* Siniperca obscura *	0	0	0	0	1	0	0	0	0	0	0
* Siniperca roulei *	1	0	0	0	1	0	0	0	0	0	0
* Siniperca scherzeri *	0	0	0	0	0	1	0	0	1	1	0
* Siniperca undulata *	0	0	0	0	0	1	0	0	0	1	0
* Odontobutis sinensis *	0	1	0	1	0	0	1	0	0	0	0
* Rhinogobius cliffordpopei *	1	1	1	1	0	0	1	0	0	1	0
* Rhinogobius duospilus *	0	1	0	0	0	0	0	0	0	0	0
* Rhinogobius giurinus *	0	1	0	1	1	1	1	1	0	1	0
* Rhinogobius lindbergi *	0	0	0	1	1	0	0	0	0	0	0
* Rhinogobius leavelli *	0	0	0	1	0	0	1	0	0	0	0
* Macropodus opercularis *	0	1	0	1	1	0	1	0	0	0	0
* Channa argus *	1	1	0	0	1	0	1	1	0	1	0
* Channa asiatica *	0	1	0	1	1	1	1	0	0	0	0
* Channa maculata *	0	0	0	0	1	1	1	0	0	0	0

## Appendix 2

**Table:** The proportion of order, family, and subfamily of fish species.

**Order**	**Species (proportion)**	**Family**	**Species (proportion)**	**Subfamily**	**Species (proportion)**
Cypriniformes	77(68.1%)	Catostomidae	1(0.9%)	Danioninae	2(3.2%)
		Cyprinidae	62(54.9%)	Leuciscinae	4(6.5%)
		Cobitidae	9(8.0%)	Culterinae	15(24.2%)
		Balitoridae	5(4.4%)	Xenocyprininae	4(6.5%)
Siluriformes	16(14.2%)	Siluridae	3(2.7%)	Hypophthalmichthyinae	2(3.2%)
		Clariidae	1(0.9%)	Gobioninae	17(27.4%)
		Bagridae	8(7.1%)	Gobiobotinae	2(3.2%)
		Amblycipitidae	3(2.7%)	Acheilognathinae	7(11.3%)
		Sisoridae	1(0.9%)	Barbinae	6(9.7%)
Beloniformes	1(0.9%)	Hemiramphidae	1(0.9%)	Cyprininae	2(3.2%)
Syngnathiformes	3(2.7%)	Syngnathidae	1(0.9%)	Labeoninae	1(1.6%)
		Mastacembelidae	2(1.8%)		
Perciformes	16(14.2%)	Percichthyidae	6(5.3%)		
		Odontobutidae	1(0.9%)		
		Gobiidae	5(4.4%)		
		Belontiidae	1(0.9%)		
		Channidae	3(2.7%)		
Total	113(100%)		113(100%)		62(100%)

## Appendix 3

**Table:** Endangered categories of fish species in the Luoxiao Mountains. Key: DD: Data Deficient; LC: Least Concern; NT: Near Threatened; VU: Vulnerable; EN: Endangered; CR: Critically Endangered.

*Species*	Endangered categories	
	Jiang et al. (2016)	IUCN (2017)
* Myxocyprinus asiaticus *	CR	DD
* Zacco platypus *	LC	DD
* Opsariichthys bidens *	LC	LC
* Mylopharyngododon piceus *	LC	DD
* Ctenopharyngodon idella *	LC	DD
* Elopichthys bambusa *	LC	DD
* Squaliobarbus curriculus *	LC	DD
* Hemiculter leucisculus *	LC	LC
* Hemiculter bleekeri *	LC	DD
* Hemiculterella sauvagei *	LC	LC
* Hemiculterella wui *	LC	DD
* Pseudohemiculter dispar *	LC	VU
* Pseudolaubuca sinensis *	LC	LC
* Sinibrama macrops *	LC	LC
* Chanodichthys erythropterus *	LC	LC
* Culter alburnus *	LC	DD
* Chanodichthys mongolicus *	LC	LC
* Chanodichthys dabryi *	LC	LC
* Culter oxycephaloides *	LC	DD
* Parabramis pekinensis *	LC	DD
* Megalobrama terminalis *	LC	DD
* Megalobrama amblycephala *	LC	LC
* Xenocypris macrolepis *	LC	LC
* Xenocypris davidi *	LC	DD
* Plagiognathops microlepis *	LC	LC
* Distoechodon tumirostris *	LC	LC
* Hypophthalmichthys molitrix *	LC	NT
* Hypophthalmichthys nobilis *	LC	DD
* Abbottina rivularis *	LC	DD
* Pseudorasbora parva *	LC	LC
* Pseudogobio vaillanti *	LC	LC
* Pseudogobio guilinensis *	LC	DD
* Hemibarbus labeo *	LC	DD
* Hemibarbus maculatus *	LC	DD
* Huigobio chenhsienensis *	LC	LC
* Sarcocheilichthys sinensis *	LC	LC
* Sarcocheilichthys kiangsiensis *	LC	DD
* Sarcocheilichthys nigripinnis *	LC	DD
* Squalidus argentatus *	LC	DD
* Rhinogobio typus *	LC	DD
* Platysmacheilus exiguus *	LC	LC
* Saurogobio dabryi *	LC	DD
* Saurogobio xiangjiangensis *	LC	DD
* Microphysogobio kiatingensis *	DD	LC
* Microphysogobio fukiensis *	DD	LC
* Gobiobotia filifer *	LC	DD
* Gobiobotia longibarba *	DD	DD
* Acheilognathus macropterus *	LC	DD
* Acheilognathus gracilis *	LC	DD
* Acheilognathus chankaensis *	LC	DD
* Acheilognathus tonkinensis *	LC	DD
* Acheilognathus barbatulus *	LC	LC
* Rhodeus ocellatus *	LC	DD
* Rhodeus lighti *	LC	LC
* Acrossocheilus fasciatus *	LC	DD
* Acrossocheilus paradoxus *	LC	DD
* Acrossocheilus hemispinus *	LC	LC
* Acrossocheilus parallens *	LC	LC
* Spinibarbus hollandi *	LC	DD
* Onychostoma barbatulum *	NT	DD
* Carassius auratus *	LC	LC
* Cyprinus carpio *	LC	VU
* Garra orientalis *	LC	LC
* Cobitis sinensis *	LC	LC
* Misgurnus anguillicaudatus *	LC	LC
* Paramisgurnus dabryanus *	LC	DD
* Schistura fasciolata *	DD	DD
* Schistura incerta *	DD	DD
* Leptobotia elongata *	VU	VU
* Parabotia banarescui *	LC	DD
* Parabotia fasciata *	LC	LC
* Parabotia maculosa *	LC	LC
* Erromyzon sinensis *	DD	DD
* Lepturichthys fimbriata *	DD	LC
* Vanmanenia stenosoma *	DD	DD
* Vanmanenia pingchowensis *	DD	DD
* Pseudogastromyzon changtingensis *	DD	DD
* Silurus asotus *	LC	LC
* Silurus meridionalis *	LC	LC
* Pterocryptis cochinchinensis *	LC	LC
* Clarias fuscus *	LC	LC
* Hemibagrus macropterus *	LC	LC
* Pseudobagrus crassilabris *	LC	DD
* Pseudobagrus tenuis *	DD	DD
* Pseudobagrus ondon *	DD	LC
* Pseudobagrus pratti *	VU	DD
* Pseudobagrus albomarginatus *	LC	DD
* Tachysurus fulvidraco *	LC	LC
* Tachysurus nitidus *	LC	DD
* Liobagrus anguillicauda *	DD	DD
* Liobagrus marginatus *	VU	DD
* Liobagrus nigricauda *	DD	EN
* Glyptothorax sinense *	LC	DD
* Hyporhamphus intermedius *	LC	DD
* Monopterus albus *	LC	LC
* Macrognathus aculeatus *	LC	DD
* Sinobdella sinensis *	DD	LC
* Siniperca chuatsi *	LC	DD
* Siniperca knerii *	LC	DD
* Siniperca obscura *	NT	LC
* Siniperca roulei *	VU	DD
* Siniperca scherzeri *	LC	DD
* Siniperca undulata *	NT	NT
* Odontobutis sinensis *	LC	DD
* Rhinogobius cliffordpopei *	LC	DD
* Rhinogobius duospilus *	DD	DD
* Rhinogobius giurinus *	LC	LC
* Rhinogobius lindbergi *	DD	DD
* Rhinogobius leavelli *	LC	LC
* Macropodus opercularis *	LC	LC
* Channa argus *	LC	DD
* Channa asiatica *	LC	LC
* Channa maculata *	LC	LC
